# Confusion around Certification of Vision Impairment (CVI) and registration processes—are patients falling through the cracks?

**DOI:** 10.1038/s41433-023-02520-0

**Published:** 2023-04-19

**Authors:** Shahina Pardhan, Robin Driscoll, Hilary Ingleton, John Slade, Michael Bowen, Rupal Lovell-Patel, Sarah Farrell, Rupert Bourne, Simon Mahoney, Sanjiv Ahluwalia, Mike Trott

**Affiliations:** 1 Vision and Eye Research Institute, Anglia Ruskin University, Cambridge, UK; 2 Royal National Institute of Blind People, London, UK; 3 College of Optometrists, London, UK; 4 NHS England and NHS Improvement, Cambridge, UK; 5 Cambridge University Hospitals NHS Foundation Trust, Addenbrooke’s Hospital, Cambridge, UK; 6 Department of Information Studies, University College London, London, UK; 7 School of Medicine, Anglia Ruskin University, Cambridge, UK; 8 Centre for Public Health, Queen’s University Belfast, Belfast, UK

**Keywords:** Health services, Quality of life

## Abstract

**Background:**

In the UK, the Certificate of Vision Impairment (CVI) certifies a person as sight impaired (partially sighted) or severely sight impaired (blind). This is completed by ophthalmologists and passed with the patient’s consent to their GP, their local authority, and The Royal College of Ophthalmologists Certifications office. Once a person is certified, they can be registered by their local authority which is voluntary but enables the person to access rehabilitation or habitation services, financial concessions, welfare benefits and other services provided by local authorities.

**Methods:**

We conducted semi-structured individual interviews with 17 patients with a diagnosed eye condition, 4 Eye Clinic Liaison Officers (ECLO) and 4 referring optometrists around their experiences around CVI and registration processes. Analysis of themes was conducted with results synthesised in a narrative analysis.

**Results:**

Patients reported lack of clarity around the processes of certification and registration, benefits of certification and what happens beyond certification, the type of support that they are entitled to, delays in accessing support. Optometrists appear not to engage with the process much, especially if the patient is being treated by the hospital eye service.

**Conclusion:**

Vision loss can be a devastating experience for the patient. There is a lack of information and confusion around the process. The lack of a joined-up process between certification and registration needs to be addressed if we are to provide the support that patients deserve in order to improve their quality of life and wellbeing.

## Introduction

In the UK, there are over 2 million people living with sight loss, with 340,000 registered blind or partially sighted. A majority (80%) are 65 years or older and well over half are women [1]. Recent trends in vision impairment in England and Wales, suggest that the rate of new CVI certifications in 2017/2018 reduced from 43 per 100,000 (2010/2011) to 41 per 100,000 an interesting finding especially in the light of an ageing population [2].

In the UK, the Certificate of Vision Impairment (CVI) certifies a person as sight impaired (partially sighted) or severely sight impaired (blind). The form is completed by ophthalmologists [3] and passed with the patient’s consent to their GP, their local authority, and The Royal College of Ophthalmologists Certifications office. NICE guidelines suggest that adults with serious eye disorders are given a certificate of vision impairment as soon as they are eligible. This may be while they are still having treatment. They should also be told about support and services, which can help them improve or regain their independence, wellbeing, and quality of life [4].

Whilst certification is carried out by the hospital eye service, support from Social Services is accessed from the patients’ local authority through Registration [5]. However, CVI is not a requirement for support from Social Services. If patients do not yet need a CVI, or if they have declined certification, they can still be referred through a Referral of Vision Impairment (RVI) [3]. In addition, optometrist can hand out a Low Vision Leaflet (LVL) for patients to self-refer instead of, or in advance of, the CVI being completed [6].

It is important to note that a CVI does not automatically register the patient for Social Services. Registration is voluntary but enables rehabilitation or habitation to be accessed, as advice around financial concessions, welfare benefits and other services provided by local authorities [6]. Social services would carry out a Social Care and Rehabilitation Assessment [7] to determine what help and advice the patient needs. This could include help with everyday tasks such as cleaning and cooking, keeping in touch with friends and family, or with transport.

Research suggests that in 2016/17, only 87.9% of people with a CVI were registered with their Local Authority, with regional variations [8]. In Hampshire, ~84% of CVIs were registered while the figure in Rutland was only 26% [8]. It is unclear whether this variation is due to personal choice, lack of knowledge about the services available, or other reasons. While social care support can lead to a greatly improved quality of life [9], studies have reported that this support is not always available. One study reported that only 51% of people with sight loss had been assessed by their local authority and only 47% reported receiving any visual impairment rehabilitation support [10]. Whether this was due to lack of engagement by the patients or lack of resources is not known. Another study found that 45% of respondents had not had the certification and registration process explained at any stage, and 41% had not received an assessment from their local authority or sensory team [11]. Long waiting times; not knowing who to contact; limited contact time with social services; lack of follow-up after a visit; and lack of resources have been reported [12].

Literature also suggests that the terms ‘Certification’ and ‘Registration’ have been used interchangeably by patients, ophthalmologists, and other healthcare practitioners (HCP), and that those certifying are not always be aware that it does not automatically lead to registration [7]^7^. It has been reported that HCPs are not always aware of the extent or type of social care offered by local authorities [13]. Differences between what is considered important by clinical staff at the hospital eye services and rehabilitation staff at social services has been reported [14].

Patient experiences of certification and registration have been extensively reviewed, with five discrete stages identified: [15]

1. Certification stage 1 – deciding it’s right to certify
2. Certification stage 2 – completing the Certificate of Visual Impairment (CVI)
3. Certification stage 3 – sending the CVI to Social Services Departments (SSDs)
4. Registration stage 1 – initial Social Services Department assessment
5. Registration stage 2 – second Social Services Department assessment

It is important to note that the sight loss pathway starts long before a patient is certified and registered—it starts when patients present themselves with an eye condition. It is important that patients are given appropriate care and advice early in the sight loss journey and signposted to support services if needed. It is also important that HCPs and in particular optometrists, who are frequently the patient’s first port of call, as well as other HCPs working in the pathways, have up-to-date knowledge around the CVI and registration processes.

The main aims of the study were to explore, using structured interviews, patient experiences around registration and certification in patients with sight loss. We also interviewed a small group of Eye Clinic Liaison Officers (ECLOs) on their experiences of patients of certification and registration processes as they have first-hand knowledge of any challenges patients may encounter during certification and registration. A small number of optometrists were also interviewed to ascertain optometrist’s knowledge around CVI, RVI and registration, and their experience when patients first present with a sight-threatening condition.

## Methods

We conducted interviews with 17 patients with eye disease, 4 community/primary care optometrists and 4 ECLOs to gain insights into the sight loss pathway in the UK. Participants were recruited purposively via external advertisements, social media, through the Royal National Institute of Blind People (RNIB) as well by contacting participants who had previously consented to be contacted for future research. All participants provided informed consent and the study was approved by the Anglia Ruskin University School of Medicine Ethics Panel (MED-SREP-21-003).

Semi-structured interviews (based on grounded theory) were conducted, with questions relating to the following: experiences of care received at different points of the sight loss pathway; experience of access to support (emotional and physical); experience of the value of information offered.

Participants were asked open-ended questions about their experiences of care across all points of their eye care pathway, their experience of certification and registration (this was intentionally worded as broadly as possible to capture the widest array of answers possible), their own personal experiences, and where they felt improvements could be made regarding certification and registration processes. Specifically, each patient was asked to describe their own experience of the process, support received, benefits from their own point of view, barriers, and what could be improved. ECLO’s were asked to describe their experiences around certification and registration. Optometrists were also asked about their knowledge on the processes and to share what their current practices were around certification, registration, and support that they think they can offer to patients.

Each interview lasted for ~30–45 min and was conducted by the same researcher (MT) to minimise inter-rater variability. Each interview was transcribed by one researcher and independently checked by another (RD, MT). Following transcription, an analysis of themes was conducted independently by two researchers (MT, RD), using NVivo (Version 12).

Following thematic analysis, the results were synthesised in a narrative analysis using a contextualist approach.

## Results

A total of 25 participants participated of which 17 were patients with varying eye conditions, 4 were referring optometrists, and 4 were ECLOs.

After analysis, three broad themes emerged:

1. A lack of clarity around the process of certification and registration:
  Patients: People with vision loss reported confusion regarding decisions and processes of certification and registration:
  ‘I was seeing a consultant who said that I wasn’t bad enough to be registered, but then his little mate in a white coat said, have you thought about getting registered? I said, I suppose I better get registered. The consultant still said, it’s not bad enough to be registered, and I thought that’s silly, (I am) too bad to drive, not bad enough to be registered, I’ve fallen down a crack here.’ (PAT5)
  ‘I did actually ask the consultant and he said no. I find it quite mystifying as I am quite impaired, I just can’t quite work out where the line is for me.’ (PAT6)
  ‘When I was born, they didn’t register me (I was blind from birth), I don’t know why. It was only later on that they realised that I should have been registered blind.’ (PAT7)
  ‘They said we really should get you registered as visually impaired, but we don’t know what’s going on, we might fix it and then getting you unregistered is going to be a bit fiddly, so let’s not do it now.’ (PAT16)
  Eye Clinic Liaison Officers: ECLO’s appeared to make individual decisions:
  ‘Without a CVI I would still make that referral [to the sensory services team] if someone’s not eligible to be registered but they still have rehabilitation needs.’ (ECLO1)
  ‘I still very much believe in the certification registration process; I think it’s a really valuable document that gives people proof who have no other way of proving something that isn’t visible to other people. I think the problem is it’s then not recognised enough in the broader society, so you’ve got the whole issue of delays within social care services and how that’s dealt with, but then you’ve got the whole issue of benefits.’ (ECLO3)
  Optometrists: A number of mixed responses were provided including not being able to make informed decisions around CVI especially if the patient is under the care of hospital:
  ‘When it comes to actual sight loss, I saw a patient eligible to be registered visually impaired. Why did I not fill in the CVI form and just get my consultant to sign it? Is it because I’m just too involved in that patient at that time that I actually forget about the whole thing?’ (OPTOM3)
2. Delays in accessing certification and support after registration:
  Patients reported long waiting lists and issues with capacity for social services support:
  ‘When it comes to sensory impairment and things like that, again it’s been a year and I’ve not heard from them.’ (PAT2)
  ‘The waiting list was very long, I remember having to wait quite some months to get that support in place.’ (PAT3)
  ECLOs’ reported delays in the process:
  ‘Well, the CVIs remain an issue. Timely CVIs. I don’t think that the medics realise that the CVI document is the gateway to services. You know it’s the evidence for people to access services and support.’ (ECLO 1)
  ‘The CVI document is the gateway to services, it’s evidence that people can access services and support. Many local authorities won’t even visit someone unless there is a CVI registration. That document is really important to actually being able to say ‘look, I need some help.’ (ECLO2)
  Optometrists provided mixed messages with regards to whether they would signpost patients for support which may delay the process:
  ‘No, because if someone is having sight loss issues, they’re referred to the hospital, and the hospital or the charities then reach out is based on what support services the doctors suggest… And so, we (optoms) don’t really trigger or get involved that much.’ (OPTOM2)
  ‘I think they don’t get much support there in some hospitals, and they leave it to the communities to do that.’ (OPTOM4)
  ‘I’ve never really ever considered referring patients to LVAs [Low Vision Assessment]’ (OPTOM3)
  ‘It is very region dependent depending on the scope and their funding.’ (OPTOM1)
3. A lack of clarity of what (and where) support can be accessed with certification and registration:

Patients gave mixed messages around the support that can be offered with registration:

‘We do feel a bit abandoned, I’m sure everybody does.’ (PAT 1)

‘No…there are no benefits socially for being certified as visually impaired such as government type things.’ (PAT1)

‘If you’re totally blind for example, the television license is half price – why you need to pay anything for a television license if you’re blind is beyond me. There are other benefits, social security benefits and what have you…but for partially sighted there’s nothing.’ (PAT1)

‘If you’re too independent they feel that you don’t need it… (as I get round fine with my guide dog, my long cane), they seem to think I shouldn’t need any support at all.’ (PAT7)

‘My sensory team in my last town were fantastic. I then transferred when I moved house, but nobody mentioned that you are supposed to transfer your sensory team. I only found out when I went out with someone from my last sensory team.’ (PAT4)

‘The consultant did tell me about it and sent me to an ECLO…. He [the ECLO] was very nice…he told me I could have a free bus pass and train tickets, nothing more than that.’ (PAT4)

‘ECLO..guided me through that process, she did a great job in terms of providing me with that support. The consultant did not give me any details of what help I would get.’ (PAT3)

‘They did say to me they could come and take me out for a walk rather than give me shopping support and I thought, well, what’s the point? I’ve got a guide dog that takes me out, I’ve got a long cane that can take me out.’ (PAT7)

‘Once I received support it was very good, and I got the support I needed to be able to travel around safely.’ (PAT3)

ECLO’s reported to inconsistencies for support offered:

‘Many local authorities won’t even visit someone unless there is a CVI in registration. That document is really important to actually being able to say ‘look, I need some help’. (ECLO 1)

‘people would come into me and say is well, I’ve been registered, I’m just waiting for XYZ and then I had to explain well you haven’t, somebody started the certificate, but it hasn’t gone anywhere (ECLO 2)

Optometrists reported on people who were not certified or registered but still needing the support:

‘There are some people who don’t qualify for the CVI or don’t want to be registered, because they haven’t got their head round the emotional barrier of being registered…but (they) still need those services. RVI is also the gateway, so it works just as well as the CVI, it doesn’t put you any lower down the waiting list or anything. It’s equal to the CVI in accessing those services. And that can be filled in by anyone in the low vision clinics, and it works equally for social services.’ (OPTOM1)

Optometrists also reported challenges when managing patients who were still registered with the hospital eye service:

‘If they’ve (patients) been to the hospital, now this is where it falls down. And a lot of them (patients) say ‘I’ve been to hospital. They say there’s nothing they can do for me.’

‘They (patients) have either registered them as partially sighted or registered as blind, and they say, ‘what do I do now? I’ve come here. Can you help me?’ And I’ve had this so many times’ (OPTOM4)

‘There needs to be so much better training (for us). We see this with the referral to social services. Traditionally we had the CVI, RVI and the LVL [Low Vision Leaflet]. So the CVI is the registration, the RVI is when the low vision clinics could refer to the rehabilitation, and the LVL is for optometrists or self-referral. I don’t know how many optometrists actually use the LVL… I don’t think many do at all and yet it doesn’t take that much time for them to do that.’ (OPTOM1)

## Discussion

Evidence from this study demonstrates confusion around the CVI, RVI and registration processes, the support patients are entitled to and offered, and how to access it from social services, charities etc. There is lack of standardisation around the CVI/RVI and registration processes which needs ‘joining up’ so that, after certification, patients can access the support they need without having to undergo further bureaucratic processes, thereby reducing the risk of them falling through the cracks and out of the system, thus missing out on vital support, services, and benefits. Data suggests that there are more patients who are certified than registered with social services [8]. Patients require more information so that they can make informed decisions about their treatment, decisions as to whether they want to be registered and/or access services even if not registered, either individually or with the help of their healthcare providers. Efficient and timely provision of information to patients about support services available locally is a clear priority and must become standard practice with anybody who is involved in the healthcare of patients.

Our study also suggests that some patients reported inconsistency amongst people who certified them, with some more inclined to certify than others. A previous study found that some ophthalmologists delayed certification due to external pressure over targets, missing people who could have been certified [15]. Previous literature shows that some healthcare practitioners may not have all the information regarding the support that can be provided by social services and, therefore, may be limited in what advice they can offer to their patients. It is also possible that ophthalmologists may be apprehensive about certifying as this could possibly be deemed as a failure in not being able to improve their patient’s visual status. A recent study showed modest agreement between thirty consultant ophthalmologists, with grading for sight impairment showing poorest levels of agreement [16].

More importantly, patients are not always aware that they can access support from the third sector without the need for certification. It is important to highlight to patients that support does not necessarily have to come after CVI. Whilst some ECLOs are aware that a CVI is not necessary for patients to access services from third sector or by social prescribers, others were not. In addition, not all NHS Trusts have ECLOs and there is a wide variation of their roles across the country [17], with some ECLOs reported to not having access to a private room, resulting in difficulties in discussing patient’s confidential situation or providing emotional support [17]. Other studies reported that just over half of ECLOs in Trusts had completed the 4-day RNIB eye clinic support studies’ course [18] which may lead to variation in the support provided [19]. In addition, patients can normally only gain access to the ECLOs services/expertise via the hospital, which is a limitation as patients may need this support much earlier in the sight loss pathway than at the point of certification.

In the absence of a community-based ECLO-type role, there is a role for optometrists to provide support in primary care settings. In our study, optometrists reported not talking to patients about support or certification generally, and there was no indication of signposting patients to charities for further support. As it is likely that some patients may require support at the earlier stages of presentation with their eye disease, it is important that everyone involved in eye health to support patients across the whole sight loss pathway, and not just after certification. Patients may benefit from earlier signposting to services that offer mental health support, rehabilitation support, or practical support, which can be accessed without the need for a CVI or RVI. Our study supports the conclusion that more timely, efficient, and effective provision of information, advice, and signposting to relevant support services in local social care and third sector organisations, along with more informed decisions around certification and registration by eye health professionals, will benefit patients.

There are a number of constraints that need to be acknowledged. Whilst we tried to ensure that the patients, ECLOs and optometrists were drawn from different parts of the UK, the scope and scale of this study constrained our ability to develop a full picture of the extent and nature of the variation across the UK. Our data showed that variation in patient experience will exist across the UK, and that that variation is likely to be driven by different approaches, understanding and attitudes to CVI/RVI and registration among HCPs, as well as the diversity in regional and local availability of social care and third sector support. In addition, the experience of ophthalmologists is missing from this study, which should be the basis for future research. Future study should include more ECLO’s and optometrists. Additional quantitative research aimed at ascertaining optometrists’ current knowledge and practices in relation to signposting patients for support without the need for a CVI across the UK, and their awareness of local services (local authority social care, and third sector), would give a better overall understanding of the landscape of primary care eye health practices. In addition, it is important that in future studies, people from under-represented groups and patients at different stages of life are included to capture varying requirements.

In conclusion, this study provided evidence that there is confusion around CVI, RVI and support generally which needs to be addressed if we are to ensure that those who lose their vision can attain the best quality of life possible.

### Summary

#### What was known before

- Whilst there is some research on how patients feel when they are certified or registered, there is little research on the extent of their awareness of the benefits and support they can access once registered.

#### What this study adds

- The study examined the experience of patients, referring optometrists and eye clinic liaison officers (ECLO’s). Patients reported lack of clarity around various process including the processes of certification and registration, benefits of certification and what happens beyond certification, the type of support that they are entitled to, delays in accessing support. Optometrists appear not to engage with the process much.

## Data Availability

Data will be available from corresponding author upon request.
